# Elevated Galectin-3 levels in the tumor microenvironment of ovarian cancer – implication of ROS mediated suppression of NK cell antitumor response via tumor-associated neutrophils

**DOI:** 10.3389/fimmu.2024.1506236

**Published:** 2024-12-20

**Authors:** Veronika Karlsson, Ebba Stål, Emma Stoopendahl, Anton Ivarsson, Hakon Leffler, Maria Lycke, Martina Sundqvist, Karin Sundfeldt, Karin Christenson, Elin Bernson

**Affiliations:** ^1^ Sahlgrenska Center for Cancer Research, University of Gothenburg, Gothenburg, Sweden; ^2^ Department of Oral Microbiology and Immunology, Institute of Odontology, Sahlgrenska Academy, University of Gothenburg, Gothenburg, Sweden; ^3^ Department of Laboratory Medicine, Lund University, Lund, Sweden; ^4^ Department of Obstetrics and Gynecology, Institute of Clinical Sciences, Sahlgrenska Academy, University of Gothenburg, Gothenburg, Sweden; ^5^ Department of Rheumatology and Inflammation Research, Institute of Medicine, Sahlgrenska Academy, University of Gothenburg, Gothenburg, Sweden

**Keywords:** galectin-3, ovarian cancer, NK cells, neutrophils, tumor immunology, ROS release, tumor microenvironment

## Abstract

**Introduction:**

Ovarian cancer is a lethal disease with low survival rates for women diagnosed in advanced stages. Current cancer immunotherapies are not efficient in ovarian cancer, and there is therefore a significant need for novel treatment options. The β-galactoside-binding lectin, Galectin-3, is involved in different immune processes and has been associated with poor outcome in various cancer diagnoses. Here, we investigated how Galectin-3 affects the interaction between natural killer (NK) cells and neutrophils in the tumor microenvironment of ovarian cancer.

**Method:**

Ascites from the metastatic tumor microenvironment and cyst fluid from the primary tumor site were collected from patients with high-grade serous carcinoma (HGSC) together with peripheral blood samples. Galectin-3 concentration was measured in ascites, cyst fluid and serum or plasma. Neutrophils isolated from HGSC ascites and autologous blood were analyzed to evaluate priming status and production of reactive oxygen species. *In vitro* co-culture assays with NK cells, neutrophils and K562 target cells (cancer cell line) were conducted to evaluate NK cell viability, degranulation and cytotoxicity.

**Results:**

High levels of Galectin-3 were observed in cyst fluid and ascites from patients with HGSC. Neutrophils present in HGSC ascites showed signs of priming; however, the priming status varied greatly among the patient samples. Galectin-3 induced production of reactive oxygen species in ascites neutrophils, but only from a fraction of the patient samples, which is in line with the heterogenous priming status of the ascites neutrophils. In co-cultures with NK cells and K562 target cells, we observed that Galectin-3-induced production of reactive oxygen species in neutrophils resulted in decreased NK cell viability and lowered anti-tumor responses.

**Conclusion:**

Taken together, our results demonstrate high levels of Galectin-3 in the tumormicroenvironment of HGSC. High levels of Galectin-3 may induce production of reactiveoxygen species in ascites neutrophils in some patients. In turn, reactive oxygen species produced by neutrophils may modulate the NK cell anti-tumor immunity. Together, this study suggests further investigation to evaluate if a Galectin-3-targeting therapy may be used in ovarian cancer.

## 1 Introduction

Ovarian carcinoma (OC) is the most lethal gynecologic malignancy among women, which can be attributed to late diagnosis of these patients and high recurrence frequency (1). The most common occurring epithelial ovarian carcinoma is high-grade serous carcinoma (HGSC), where many women are diagnosed at an advanced stage (Federation of Gynecology and Obstetrics [FIGO] stage III or IV) (2, 3). Current treatment strategy includes debulking surgery followed by platinum-based chemotherapy. However, as recurrence occurs in 70% of the patients, novel treatment options are of significant clinical need. Immunotherapy has emerged as a successful treatment option in several cancers but has not yet been proven as an efficient treatment for OC (4, 5). To improve or develop novel treatment options for OC patients, a better understanding of the immune microenvironment in OC is urgently needed. OC metastasis is commonly followed by the accumulation of fluid, or ascites, in the peritoneal cavity. The ascites contains both malignant cells, lymphocytes and granulocytes, and acellular components such as interleukin (IL)-6, IL-8, IL-10, transforming growth factor beta (TGF-ß) and vascular endothelial growth factor (VEGF) (6–8). The correlation between T cell infiltration to the OC tumor microenvironment and improved survival suggests that immunotherapy is a conceivable treatment option in the disease (9). However, despite a correlation between mutational burden and immune cell tumor infiltration, many OC tumors remain immunologically “cold” and do not evoke a specific T cell response (10). Thus, OC immunotherapy directed towards targets beyond cytotoxic T cells may present as a promising alternative.

Natural killer (NK) cells are innate lymphocytes that, in contrast to cytotoxic T cells, have the ability to detect and kill malignant cells without prior sensitization. Upon activation, NK cells exert their cytotoxic function through degranulation of lytic granules containing pore-forming proteins and proteases (11–13). We have recently demonstrated that a subset of tissue-resident NK cells in OC ascites display anti-tumor properties, suggesting that NK cells are a feasible immunotherapeutic target (7). However, the NK cell function in OC is impaired due to the immunosuppressive tumor microenvironment (4, 8, 14–17).

An immune-suppressor that has gained interest in cancer research is Galectin-3, a mammalian lectin with affinity for *β*-galactoside-containing glycoconjugates. Galectin-3 is involved in a number of different biological processes including inflammation, apoptosis, cell growth and angiogenesis (18–24). Blood levels of Galectin-3 are often increased during inflammatory conditions (18), however, high Galectin-3 blood levels have also been detected in several cancers including colon, head and neck, liver, gastric, endometrial, thyroid, skin, bladder and breast carcinomas (25, 26). Galectin-3 may promote tumorigenesis and metastasis through several mechanisms including induction of T cell apoptosis, inhibition of tumor cell apoptosis, promotion of angiogenesis, adhesion between tumor and endothelial cells, and promotion of tumor spread (25–27). Indeed, increased Galectin-3 blood levels have been associated with bad prognosis and/or relapse in breast, lung, and oral cancer (28–31). In OC, Galectin-3 can be detected on the cell surface of primary tumor cells and OC cell lines. Moreover, Mirandola et al. have demonstrated that inhibition of Galectin-3 reduces growth, invasion, migration, and drug resistance of OC cells *in vitro*, and interferes with the angiogenic potential of OC cells (27). Currently, Galectin-3 inhibition is being evaluated as potential treatment in both malignant and non-malignant conditions (25, 26).

Neutrophils are one type of immune cell present in OC ascites (32). Neutrophils are important effector cells in the first line of defense and eliminate pathogens through phagocytosis, degranulation of vesicles containing toxic and proteolytic factors, release of reactive oxygen species (ROS) and formation of neutrophil extracellular traps (NETs) (20, 33). While neutrophils circulating in peripheral blood are in a resting state, extravasation to tissue usually results in a switch to a pre-activated, primed, state. Priming of neutrophils commonly includes degranulation of intracellular granules and secretory vesicles, which can be characterized as cleavage of surface-bound L-selectin (CD62L) and upregulation of granule localized receptors on the cell surface, including CD11b and CD66 (34–36). Secretory vesicles are most easily mobilized to the plasma membrane followed by gelatinase and specific granules; the granules requiring the most stimuli for mobilization are the azurophilic granules. Depending on the extent of stimuli, granule membrane-localized receptors are thus exposed on the neutrophil cell surface and matrix localized soluble factors are released to the extracellular environment (37, 38). Interestingly, degranulation of the gelatinase and specific granules exposes Galectin-3 binding sites on the surface of neutrophils that allow stimulation of the NADPH oxidase, as Galectin-3 stimulates ROS release in neutrophils extravasated into tissue or *in vitro* treated with TNF-α, but not in resting neutrophils from the blood circulation (39, 40).

ROS released by myeloid cells has been correlated to decreased NK cell cytotoxicity against myeloid leukemia cells (41). We hypothesized that Galectin-3 mediated ROS released from extravasated neutrophils in the metastatic environment of OC ascites would affect NK cell anti-tumor responses. Thus, in this study, we investigated the impact of Galectin-3 on the interaction between neutrophils, NK cells and tumor cells. Our data demonstrated the presence of soluble Galectin-3 in the primary and ascitic HGSC tumor microenvironment, together with the presence of degranulated neutrophils. We observed that the extent of degranulation varied among patient samples, and Galectin-3-induced ROS production by ascites neutrophils was apparent in a fraction of patients with HGSC. Using functional NK cell anti-tumor assays, we investigated how Galectin-3-induced ROS release from neutrophils impacted on NK cell functionality. Our results demonstrated a Galectin-3 mediated decrease of NK cell viability *via* neutrophil ROS release, with anti-tumor responses impeded by ROS.

## 2 Materials and methods

### 2.1 Patients and sample collection

This study includes biosamples from two cohorts of patients with confirmed or suspected HGSC. Ascites, cyst fluid and blood samples were collected from Cohort 1 (sampled during 2016), and ascites and blood samples were collected from Cohort 2 (sampled during 2020-2024). Sampling was carried out during de-bulking surgery or paracentesis prior to surgery at Sahlgrenska University Hospital, Gothenburg, Sweden, after informed written consent from patients. Only chemo naïve patients were included in the study. All histopathology evaluation was performed by a board-certified pathologist specializing in gynecological malignancies. Patient and tumor data were recorded regarding age, body mass index (BMI), smoking and FIGO stage (summarized in **Tables 1**, **2**). Ten patients with HGSC were enrolled in Cohort 1 and 18 patients with HGSC were enrolled in Cohort 2. The studies were approved by the regional ethics board in Gothenburg (Dnr. 201-15) and the Swedish Ethical Review Authority (Dnr. 510-13) and performed according to the Helsinki declaration. Buffy coats and blood from healthy donors were obtained from the blood bank at the Sahlgrenska University Hospital, Gothenburg, Sweden. As the buffy coats and blood were provided anonymously and thereby could not be traced back to a specific donor, no ethical approval was needed in accordance with the Swedish legislation section code 4§ 3p SFS 2003:460 (Law on Ethical Testing of Research Relating to People).

**Table 1:** Patient characteristics in Cohort 1.

Patient ID	Stage^a^	Age^b^	BMI^c^	Smoking^d^	Ascites Galectin-3 conc. (ng/mL)	Cyst fluid Galectin-3 conc. (ng/mL)	Serum Galectin-3 conc. (ng/mL)
1	IIIC	52	23	No	31.9	112.3	6.2
2	IIIC	40	21	No	110.4	111.9	7.2
6	IIIC	57	19.5	No	15.5	–	10.9
7	IIIC	64	20.6	No	15.8	115.3	9.8
10	IIIC	69	26.5	No	35.4	92.1	28.0
13	IVB	68	22.1	No	19.8	60.0	4.9
14	IVB	58	25	No	70.4	–	7.4
15	IIIC	48	23.9	No	26.4	82.6	8.4
18	IIIC	71	22.1	No	12.3	–	10.7
20	IIB	60	18.5	No	87.4	20.4	27.4

^a^FIGO stage ^b^Age in years ^c^Body mass index ^d^Yes or no.

**Table 2:** Patient characteristics in Cohort 2.

Patient ID^a^	Stage^b^	Age^c^	BMI^d^	Smoking^e^	Ascites volume (L)	Ascites Galectin-3 conc. (ng/mL)	Plasma Galectin-3 conc. (ng/mL)
4	IIIC	41	25.8	No	1.3	10.2	14.2
10	IIIC	73	27.1	No	1.3	32.9	10.7
12	IV	66	31.1	No	≥ 2	35.8	16.5
13	IIIA1	64	19.8	No	≥ 2	14.9	13.6
16	IIB	51	28.9	No	≥ 2	64.7	18.3
24	IVB	58	32.3	No	≥ 2	20.5	38.5
26	IIIC	46	33.9	No	≥ 2	16.8	12.4
28	IIIC	77	31.2	No	≥ 2	25.5	24.4
29	IIIC	65	20.4	No	0.2	64.2	17.7
30	IIIC	70	28.5	No	0.65	24.8	9.3
33	IIIC	55	39.4	Yes	≥ 2	55.1	18.8
35	IVB	60	41.3	No	0.57	44.2	28.6
36	IIIC	58	32.3	No	0.85	32.2	9.1
40	IIIC	60	18.8	No	1.5	8.3	16.4
45	IVB	50	23.4	No	0.4	145.8	14.4
48	IVB	52	19.9	No	1.9	34.5	20.5
49	IVA	65	24.7	No	≥ 2	40.6	19.4
54	IIIC	61	20.5	No	≥ 2	15.9	17.4

^a^Patients within this cohort were included in one earlier publication (7). ^b^FIGO stage. ^c^Age in years. ^d^Body mass index. ^e^Yes or no.

### 2.2 Biosample preparation

Cohort 1: Cyst fluid, taken from surgically excised ovarian cysts, and ascites, aspirated at the time of midline incision, were collected in silicon dioxide tubes and frozen at -80°C within 4 hours after collection. Venous blood was collected in silicon dioxide tubes and centrifuged, after which serum was transferred to new tubes and stored at -80°C.

Cohort 2: Venous blood was collected in EDTA tubes. For plasma collection, blood was centrifuged at 1000 x *g* (10 min, 4°C), after which plasma was transferred to new tubes and frozen at -80°C. Ascites was aspirated either at the time of midline incision, or through paracentesis, and collected in plastic collection bags. Cell-free ascites was obtained by centrifugation at 1000 x *g* (10 min, 4°C). Ascites was filtrated using 180 and 40 µm nylon net filters (Merck Millipore) to achieve a single cell suspension.

Erythrocytes were removed from ascites, venous blood and buffy coats with dextran sedimentation. This was followed by density gradient centrifugation with lymphoprep (STEMCELL Technologies) to obtain mononuclear cells and neutrophils. NK cells were isolated from the mononuclear cells using a negative NK isolation kit (Miltenyi Biotec) according to manufacturer’s protocol. The neutrophils, obtained in the lymphoprep pellet, were treated with distilled H_2_O to remove remaining erythrocytes by hypotonic lysis and then stored in Krebs-Ringer Glucose phosphate buffer (KRG; pH 7.3, supplemented with Ca^2+^ [1 mM]) on ice prior to subsequent analysis on the same day as isolated. For cell morphology and phenotype after isolation, see **Supplementary Figures S1A, C**. For some experiments the neutrophils were pre-treated with recombinant human TNF-α (10 ng/ml; Sigma-Aldrich) for 20 min at 37°C; neutrophils used as controls to these cells were kept on ice. When specified, blood neutrophils were incubated in cell-free cyst fluid (Cohort 1) or autologous cell-free ascites (Cohort 2) for 20 min at 37°C; neutrophils used as controls were incubated in KRG for 20 min at 37°C. Serum from healthy donors was obtained from venous blood by centrifugation and stored at -80°C.

### 2.3 Measurement of soluble Galectin-3

Paired biosamples of ascites, cyst fluid and serum (Cohort 1), or ascites and plasma (Cohort 2), from patients with confirmed HGSC and serum from age-matched healthy donors were analyzed for content of Galectin-3 using an enzyme-linked immunosorbent assay (ELISA) from BG Medicine according to the protocol provided by the manufacturer. The total protein concentration in the biosamples was determined by Pierce BCA Protein Assay (Thermo Scientific) according to manufacturer’s instructions. Absorbance for Galectin-3 concentration was measured at 450 nm in a CLARIOstar plate reader (BMG Labtech), while absorbance for total protein concentration was measured at 562 nm in a FLUOstar Omega plate reader (BMG Labtech). Results were calculated in Microsoft Excel version 16.57 or later.

### 2.4 Detection of cell-bound Galectin-3

Cell-bound galectin-3 was analyzed on non-isolated leukocytes. Filtrated single cell ascites and blood from cohort 2 were treated with FACS lysing solution (BD FACS) according to manufacturer’s instructions on the day of biosample collection. Thereafter, the fixated leukocytes were washed with PBS and immunostained for one hour at 4°C in darkness. Antibodies were diluted in PBS supplemented with 10% human serum. Neutrophils were distinguished from other leukocytes with BV786 anti-CD45 monoclonal antibody (clone HI30; BD Horizon) and light scattering, see **Supplementary Figure S1B** for gating. Galectin-3 binding was detected using PE anti-Galectin-3 monoclonal antibody (clone M3/38) with a matching isotype control (PE rat IgG2A, κ antibody), both purchased from BioLegend. All flow cytometry analysis in this study was performed using LSRFortessa (BD) and data were analyzed using FlowJo version 10.8.2 or later (BD Biosciences). Results are shown as median fluorescence intensity (MFI) if not stated otherwise.

### 2.5 Phenotypic analysis of neutrophil priming status

The priming status of neutrophils were phenotypically examined by analyzing the expression of CD11b, CD66, CD66b and CD62L on the cell surface by flow cytometry. Neutrophils were either kept on ice or pre-treated with TNF-α or incubated with cell-free ascites/cyst fluid (as described above), and thereafter washed in KRG and immunostained for 30 min at 4°C in darkness. The following fluorochrome-conjugated monoclonal antibodies were used for detection of surface markers: APC anti-CD11b (clone ICRF44), PE anti-CD66a,c,d,e (clone B1.1/CD66), FITC anti-CD66b (clone G10F5) and APC anti-CD62L (clone DREG-56), all antibodies were purchased from BD Pharmingen.

### 2.6 Production of recombinant Galectin-3

Recombinant human Galectin-3 was produced in *E. coli* and purified as previously described (39, 42). For some experiments Detoxi-Gel Endotoxin Removing Columns (Thermo Scientific) were used for endotoxin removal of Galectin-3 according to protocol provided by the manufacturer.

### 2.7 Measurement of neutrophil ROS production

Production of ROS by the neutrophil NADPH oxidase was measured with an isoluminol-amplified chemiluminescence system in the presence of horse radish peroxidase as described by Dahlgren et al. (43). Resting and TNF-α treated neutrophils were diluted in KRG and equilibrated in polypropylene tubes (1 ml system with 1 x 10^5^ cells) in a six-channel Biolumat LB 9505 (Berthold Technologies) or in white 96-well plates (0.2 ml system with 5 x 10^5^ cells) CLARIOstar plate reader (BMG Labtech) for 5 min at 37°C, with or without lactose (10 mM, Sigma Aldrich). After equilibration, cells were stimulated with recombinant human Galectin-3 (20 µg/ml), formyl-methionyl-leucyl-phenylalanine (fMLF; 100 nM, Sigma Aldrich) or phorbol 12-myristate 13-acetate (PMA; 50 nM, Sigma Aldrich) and the light emission, which reflect the superoxide anion production (the pre-cursor of all ROS), was recorded over time. The ROS levels measured using the Biolumat LB 9505 are expressed as mega counts per minute (Mcpm) and the ROS levels measured using the CLARIOstar plate reader are expressed as relative light units (RLU). For analysis of peak ROS values, the background level, i.e., the value recorded prior to stimulation was subtracted from the observed peak value. The results were analyzed using GraphPad Prism software version 10.2.0 or later (GraphPad Software).

### 2.8 NK cell viability, degranulation and cytotoxicity

NK cells and neutrophils were isolated from buffy coats from healthy donors using negative beads as described above. The human myelogenous leukemia cell line, K562, was provided by the Department of Infectious Diseases at the University of Gothenburg, Sweden. Complete medium used in assays contained RPMI 1640 (Gibco) and 10% heat-inactivated fetal calf serum. Neutrophils were pre-treated with TNF-α as described above. NK cells and neutrophils were co-incubated in a low attachment 96 well plate (Corning) at 1:1 or 2:1 ratio (NK:neutrophils) in medium only or with endotoxin free Galectin-3 (5 or 25 µg/ml), with or without the addition of SOD (50 U/ml, Worthington Biochemical) and endotoxin free catalase (200 U/ml, Worthington Biochemical), or diphenyleneiodonium (DPI; 3 µM, Sigma Aldrich), for 4 hours at 37°C with 5% CO_2_. After 4 hours K562 cells, pre-labeled with CellTrace Violet (Invitrogen) according to manufacturer’s protocol, were added at a 5:1 ratio (NK:K562) followed by BUV395 anti-CD107a monoclonal antibody (clone H4A3, BD Horizon) to measure NK cell degranulation, and then incubated for 20 hours at 37°C with 5% CO_2_. Cells were labeled with BV711 anti-CD56 monoclonal antibody (clone NCAM 16.2, BD Horizon), for NK cell identification, and LIVE/DEAD Fixable Near-IR (1:1000, Invitrogen) to measure cell viability. Gating strategies are provided in **Supplementary Figure S1D**. The data was analyzed using flow cytometry as described above.

### 2.9 Statistical analysis

Statistical analysis was performed in GraphPad Prism version 10.2.0 or later. For comparison of two groups a Student’s t-test was used. For multiple group comparisons with paired data, repeated measures one-way ANOVA or mixed-effects model using restricted maximum likelihood (REML) estimation was used. The mixed-effects model using REML estimation was used instead of one-way ANOVA when there were missing values in the data sets. For multiple group comparisons with no pairing, ordinary one-way ANOVA was used. More detailed information about the statistical tests used in each experiment is stated in figure legends. Statistically significant differences were regarded as *p*-values < 0.05.

## 3 Results

### 3.1 Galectin-3 is present in the HGSC tumor microenvironment

**Figure 1:**
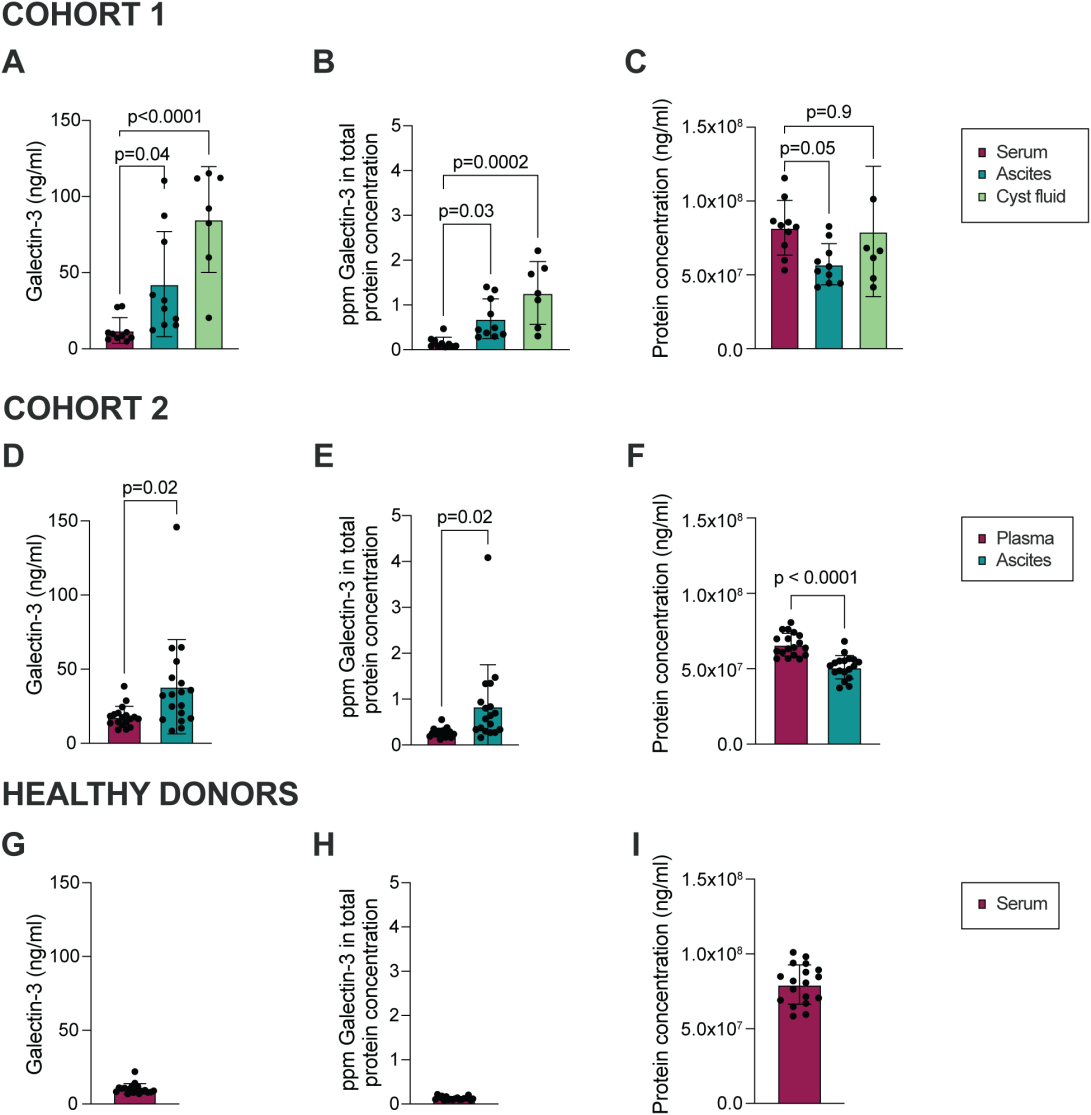
Galectin-3 is significantly higher in HGSC ascites and cyst fluid as compared to serum. **(A)** Galectin-3 concentration in ascites, cyst fluid and serum collected from patients with HGSC within Cohort 1. **(B)** Concentration of Galectin-3 in serum, ascites and cyst fluid normalized to total protein concentration, shown as parts per million (ppm; Cohort 1). **(C)** Total protein concentration in matched ascites, serum and cyst fluid (Cohort 1). **(D)** Galectin-3 concentration in ascites and plasma collected from patients with HGSC within Cohort 2. **(E)** Concentration of Galectin-3 in plasma and ascites when normalized to total protein concentration (ppm; Cohort 2). **(F)** Total protein concentration in matched ascites and plasma (Cohort 2). **(G)** Galectin-3 concentration in serum collected from healthy donors. **(H)** Concentration of Galectin-3 in serum when normalized to total protein concentration (ppm; healthy donors). **(I)** Total protein concentration in serum (healthy donors). Data is presented as mean ± SD, and statistically significant differences were evaluated by REML mixed-effects model followed by Šídák’s multiple comparisons test **(A-C)** or paired Student’s t-test **(D-F)**, n=7-10 in Cohort 1, n=18 in Cohort 2, n=18 in healthy donor cohort.

Galectin-3 levels in ascites from 10 patients diagnosed with HGSC (Cohort 1, **Table 1**) were investigated using ELISA. The mean Galectin-3 concentration was significantly higher in ascites compared to paired serum samples from HGSC patients (**Figure 1A**; median 9.1 and 29.1 ng/ml in serum and ascites, respectively). Also, ovarian cyst fluid collected from the primary tumor site contained increased levels of Galectin-3 as compared to paired serum (median 92.1 ng/ml in cyst fluid). To verify that the higher level of Galectin-3 measured in ascites was not due to an increase in total protein concentration, the Galectin-3 level was normalized to total protein concentration. The relative Galectin-3 concentration (fraction of total protein concentration) was significantly higher in ascites compared to serum (**Figure 1B**; total protein concentration in ascites, serum and cyst fluid are displayed in **Figure 1C**). We repeated the analysis with samples from 18 patients with HGSC (Cohort 2, **Table 2**) and the results verified that the level of Galectin-3 was increased in HGSC ascites as compared to paired plasma (**Figures 1D–F**; median 16.9 and 32.6 ng/ml in plasma and ascites, respectively). To investigate if the increased levels of Galectin-3 were local or systemic, we measured the concentration of Galectin-3 and total protein concentration in serum from age-matched healthy donors (**Supplementary Table S1**, **Figures 1G–I**; median 9.1 ng/ml). Serum and plasma levels of Galectin-3 were similar between the two HGSC cohorts and healthy donors, suggesting that the increased levels of Galectin-3 are not systemic but localized to the tumor microenvironment.

### 3.2 Neutrophils in ascites from HGSC patients show signs of priming

**Figure 2:**
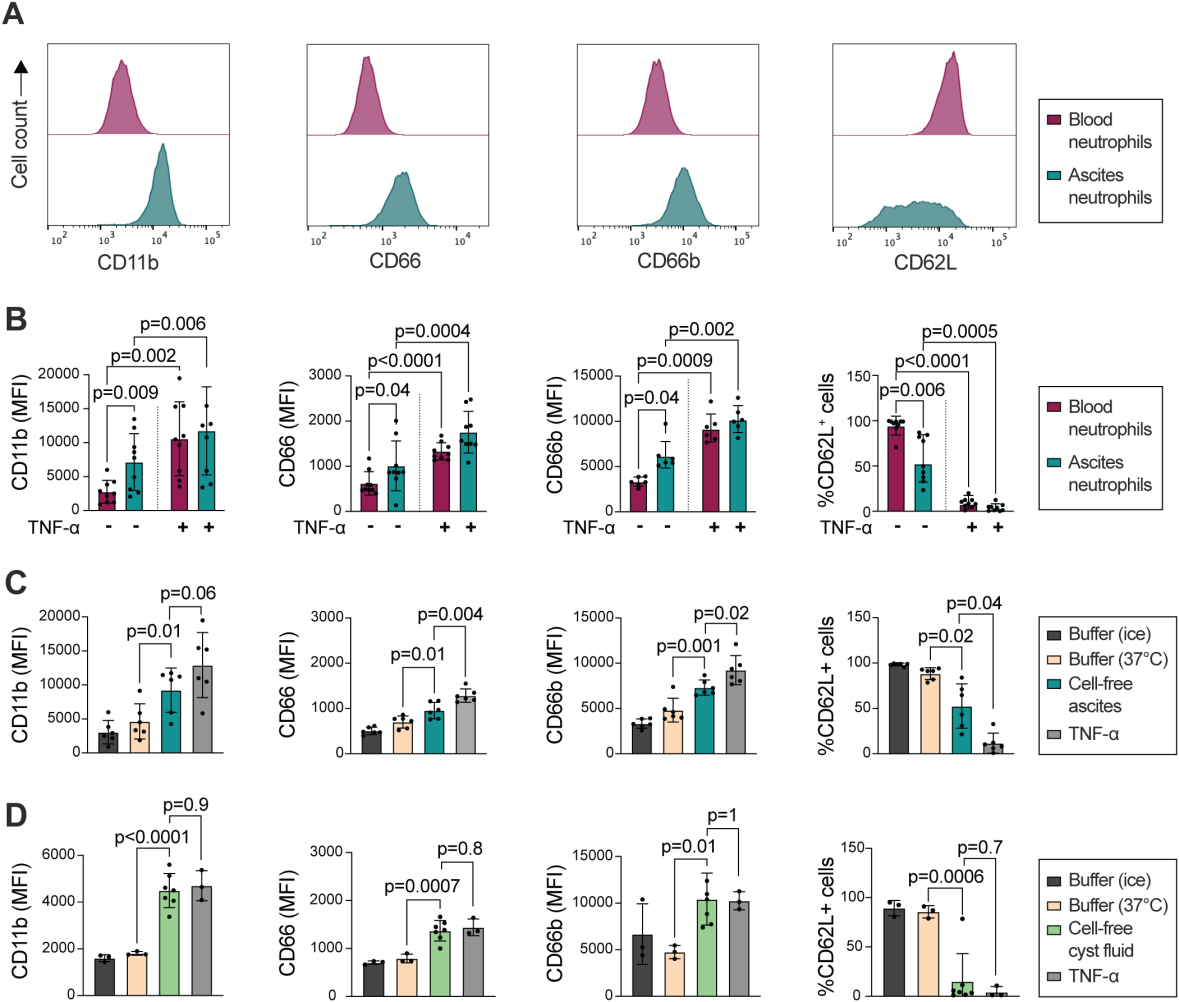
HGSC ascites neutrophils show signs of priming compared to autologous peripheral blood neutrophils. **(A)** Representative histograms of surface expression of CD11b, CD66, CD66b and CD62L. **(B)** Surface expression of CD11b, CD66, CD66b (MFI), and percentage of CD62L^+^ neutrophils, on HGSC peripheral blood neutrophils (red) and HGSC ascites neutrophils (green) on unstimulated and TNF-α treated cells. **(C)** Surface expression of CD11b, CD66, CD66b (MFI), and percentage of CD62L^+^ neutrophils, on HGSC peripheral blood neutrophils, after incubation in autologous cell-free HGSC ascites, buffer (KRG) or TNF-α at 37°C for 20 min, or kept in KRG on ice. **(D)** Surface expression of CD11b, CD66, CD66b (MFI), and percentage of CD62L^+^ neutrophils, on healthy donor peripheral blood neutrophils that were incubated in cell-free HGSC cyst fluid, buffer (KRG) or TNF-α at 37°C for 20 min, or kept in KRG on ice. Blood neutrophils from 3 healthy donors were incubated in cell-free cyst fluid from 7 patients with HGSC. Data is presented as mean ± SD, and statistically significant differences were evaluated with repeated measures one-way ANOVA followed by Šídák’s multiple comparisons test **(B-C)** and ordinary one-way ANOVA followed by Šídák’s multiple comparisons test **(D)**, n=6-9 in **(B)**, n=6 in **(C)**, n=3/7 in **(D)**.

Neutrophil response to stimuli is dependent on their priming status; resting peripheral blood neutrophils will respond differently to activating agonists as compared to extravasated (primed) neutrophils at the site of inflammation (44). As previously shown by others, Galectin-3 stimulates ROS production in primed, but not resting, neutrophils (39). Priming is most often associated with degranulation and subsequent alterations of receptors on the neutrophil cell surface. Similarly, *in vitro* treatment with TNF-α results in primed neutrophils, with altered surface expression (increased CD11b and CD66 and decreased CD62L), as well as a responsiveness to Galectin-3-induced ROS-release (33, 40). Therefore we used flow cytometry to investigate the cell surface expression of CD62L (L-selectin), CD11b (integrin alpha M), CD66a,c,d,e (hereafter referred to as CD66) and CD66b, surface markers characteristic for primed neutrophils, on HGSC ascites neutrophils as compared to autologous peripheral blood neutrophils. As shown in **Figures 2A, B**, surface expression of CD11b, CD66 and CD66b was upregulated on HGSC ascites neutrophils, while CD62L was shed, in comparison to blood neutrophils, indicating that neutrophils present in HGSC ascites display a primed phenotype. However, treatment with TNF-α *in vitro* led to additionally increased expression of CD11b, CD66 and CD66b, as well as lower expression of CD62L, which suggests that HGSC ascites neutrophils can be further primed (**Figure 2B**). The surface expression of CD11b, CD66 and CD66b, and percentage of CD62L^+^ neutrophils, observed in blood neutrophils isolated from healthy donor buffy coats (**Supplementary Figures S2A–D**) was similar to blood neutrophils from OC patients, indicating priming of neutrophils in the tumor environment specifically.

We hypothesized that in addition to transmigration to the peritoneum, neutrophil priming may be induced by the tumor microenvironment in HGSC. Thus, we next tested whether cell-free ascites or cyst fluid from patients with HGSC could prime resting blood neutrophils. Peripheral blood neutrophils were incubated in autologous cell-free ascites or cyst fluid followed by measurement of cell surface expression of CD62L, CD11b, CD66 and CD66b. As shown in **Figures 2C, D**, peripheral blood neutrophils have higher expression of CD11b, CD66 and CD66b, and less expression of CD62L, on the cell surface after incubation in autologous HGSC cell-free ascites or cyst fluid. In relation to the priming induced by TNF-α treatment, neutrophils incubated in cell-free cyst fluid displayed a higher extent of priming compared to neutrophils incubated in cell-free ascites. Together, the results indicate that acellular components in the HGSC tumor microenvironment induces priming in neutrophils.

Upon priming, during the degranulation process, neutrophils expose additional Galectin-3-binding sites (39, 40). We thus investigated the level of Galectin-3 on the cell surface on HGSC ascites neutrophils. The amount of surface-bound Galectin-3 varied between samples, and we observed a trend of increased Galectin-3 bound to ascites neutrophils when compared to autologous peripheral blood neutrophils in two out of four patient samples, however the increase was not significant (**Supplementary Figure S2E**).

### 3.3 Galectin-3-induced ROS release in HGSC ascites neutrophils differ among patients

**Figure 3:**
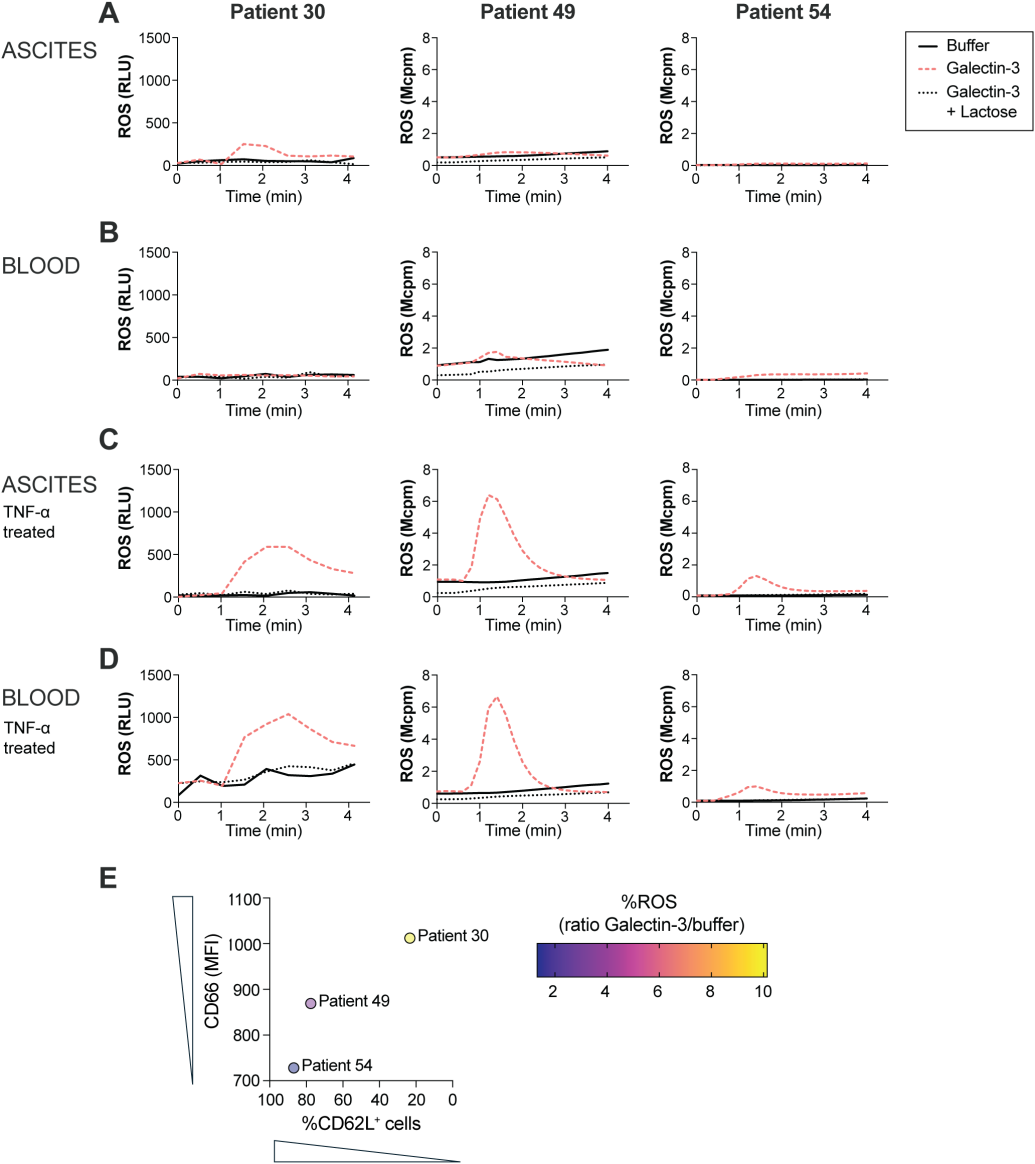
ROS release in ascites and peripheral blood neutrophils from three HGSC patients. **(A-D)** ROS release upon exposure to Galectin-3 (20 µg/ml) in untreated ascites **(A)** and blood **(B)** neutrophils, and TNF-α treated ascites **(C)** and blood **(D)** neutrophils in the presence or absence of lactose (10 mM). **(E)** Correlation between ROS release as measured by ratio of ROS peak value between ascites neutrophils stimulated with Galectin-3 and unstimulated (buffer) ascites neutrophils, and priming status as measured by surface expression of CD66 and percentage of CD62L^+^ neutrophils, in HGSC ascites neutrophils from 3 patients with HGSC. ROS release in neutrophils from patient 30 was measured using CLARIOstar plate reader, while ROS release in neutrophils from patient 49 and 54 was measured using Biolumat LB 9505.

Earlier studies have shown that Galectin-3 induces ROS release in *in vivo* extravasated neutrophils and in neutrophils treated *in vitro* with ionomycin, fMLF, TNF-α or lipopolysaccharide (LPS) (39, 40, 45–47), while resting neutrophils, or neutrophils modestly stimulated in 22°C, did not respond to Galectin-3 with ROS release (39). The results from the study by Karlsson et al. imply that a certain amount of intracellular granules needs to be mobilized to the surface in order for a neutrophil to be able to respond with ROS release upon Galectin-3 stimulation. As OC ascites neutrophils displayed a primed phenotype, we next investigated whether Galectin-3 induces ROS release in these cells without prior TNF-α treatment *in vitro*. **Figures 3A, B** shows ROS release in ascites and peripheral blood neutrophils from three HGSC patients. While *in vitro* TNF-α treated neutrophils, from both HGSC ascites and peripheral blood, released ROS upon Galectin-3 stimulation (**Figures 3C, D**), only one patient sample of ascites neutrophils that were not pre-exposed to TNF-α *in vitro* responded to Galectin-3 with release of ROS with similar kinetics to Galectin-3-induced ROS release in TNF-α treated neutrophils (patient 30; **Figures 3A–D**). The ROS release curves from HGSC ascites neutrophils from the two other samples (patients 49 and 54) were similar to unstimulated peripheral blood neutrophils after Galectin-3 stimulation, with no or neglectable ROS release detected. When the Galectin-3 inhibitor lactose was added to the neutrophils, no Galectin-3 induced ROS was detected in any of the patient samples (**Figures 3A–D**). Both ascites and blood neutrophils produced ROS after stimulation with PMA, and neutrophils from patients 49 and 54 also produced ROS after stimulation with fMLF (**Supplementary Figure S3**; neutrophils from patient 30 were not stimulated with fMLF). To understand why the ascites neutrophils responded differently across the patient samples, we plotted the ROS response to Galectin-3 against the priming status of the ascites neutrophils. As demonstrated in **Figure 3E**, the HGSC ascites neutrophils that released the highest amount of Galectin-3-induced ROS were also the most primed neutrophils (as measured by surface expression of CD66 and percentage of CD62L^+^ neutrophils), however we had too few samples to statistically correlate the parameters to each other.

### 3.4 Galectin-3-induced ROS production decreases NK cell viability *in vitro* and impairs NK cell function against tumor cells

**Figure 4:**
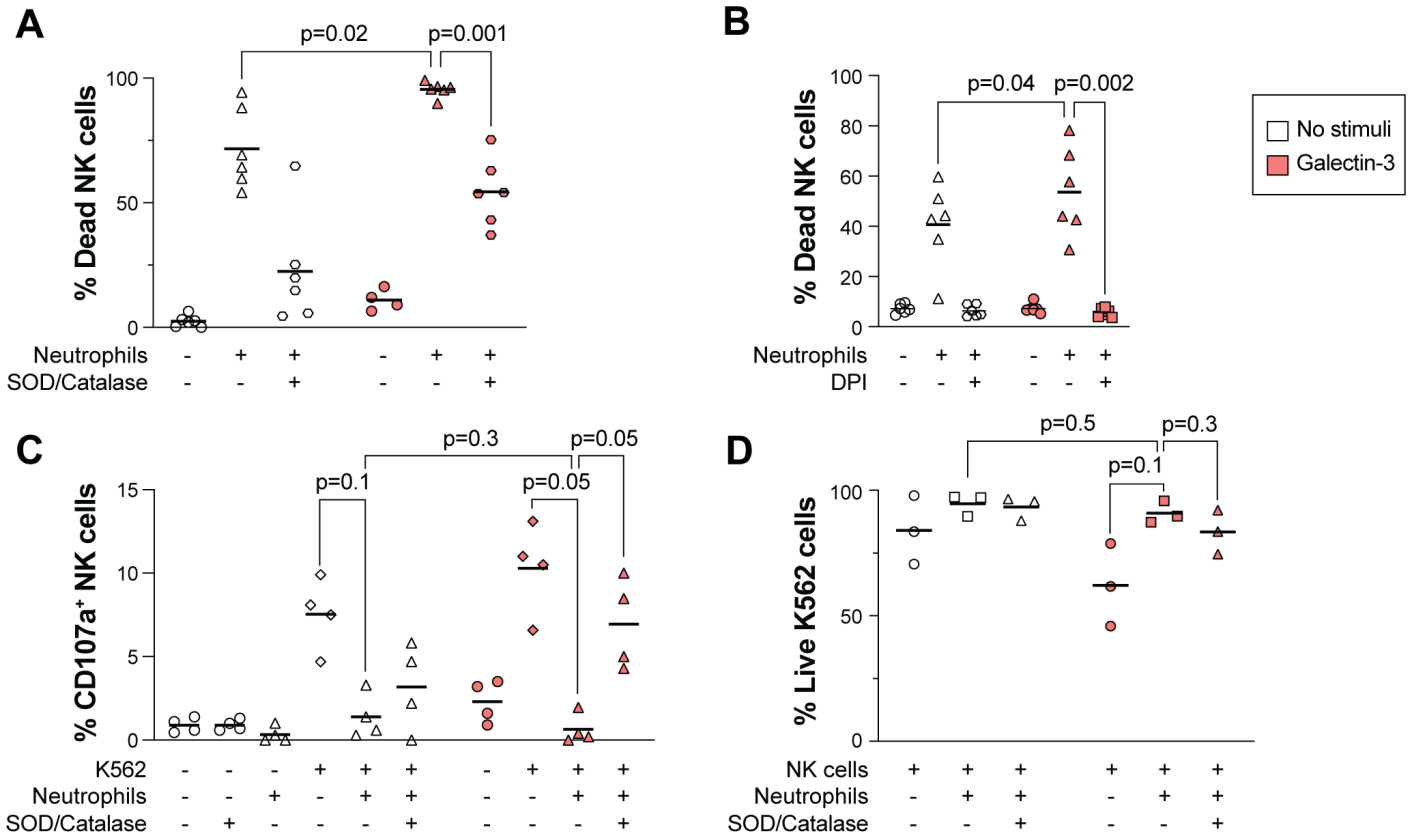
Galectin-3-induced ROS production in neutrophils decreases NK cell viability. **(A, B)** Impact of Galectin-3 on NK cell viability in presence or absence of neutrophils, measured as percentage of dead NK cells. NK cells and TNF-α treated neutrophils were co-incubated at a 2:1 **(A)** or 1:1 **(B)** ratio in medium only (no stimuli) or with Galectin-3 (25 or 5 µg/ml in A and B, respectively) overnight, with or without the addition of SOD and catalase **(A)** or DPI **(B)**. **(C, D)** NK cell degranulation as measured by CD107a expression **(C)** and viability of K562 cells **(D)** after overnight co-culture with NK cells, K562 cells and TNF-α treated neutrophils. Galectin-3 (25 µg/ml) and SOD/catalase were added as indicated. Horizontal lines represent mean. Statistically significant differences were evaluated by repeated measures one-way ANOVA followed by Šídák’s multiple comparison test **(A-D)**, n=6 in **(A, B)**, n=4 in **(C)**, n=3 in **(D)**.

Exposure to ROS decreases the NK cell viability (41). We thus hypothesized that Galectin-3-induced ROS release in neutrophils may impact NK cell viability and hence their anti-tumor function. As shown in **Figures 4A, B**, co-incubation with NK cells and neutrophils resulted in decreased NK cell viability, which was further decreased when Galectin-3 was added to the setup. The addition of DPI, a known NADPH oxidase inhibitor, or SOD and catalase that acts as ROS scavengers, restored the NK cell viability, indicating that the observed NK cell death is mediated *via* a ROS-mediated mechanism.

We next evaluated the NK cell anti-tumor effect in a co-culture assay with NK cells and target cells from the leukemic tumor cell line K562, an established model to measure NK cell reactivity towards tumor cells (48). Upon NK cell activation after interaction with ligands on target cells, NK cells release lytic granules containing Granzyme B and perforin that kill the target cell (49). In the presence, but not absence, of Galectin-3, addition of neutrophils to the co-culture decreased the NK cell degranulation significantly, as measured by percentage of NK cells expressing CD107a (**Figure 4C**). This was paralleled with a trend of increased viability of K562 cells, however the difference was not significant (**Figure 4D**; gating strategy in **Supplementary Figure S1D**). The addition of SOD and catalase to the assay restored NK cell degranulation, indicating that ROS-mediated NK cell death was responsible for their decreased anti-tumor activity (**Figure 4C**).

## 4 Discussion

In this study, we have investigated how the presence of Galectin-3 in high-grade serous carcinoma (HGSC) affects the immune-mediated anti-tumor response. Using samples from patients with HGSC we detected high levels of Galectin-3 in the ascites together with degranulated neutrophils. Furthermore, our *in vitro* functional assays with NK cells, neutrophils and tumor cells demonstrated a decreased NK cell response in the presence of Galectin-3. Taken together, the results from this study imply that Galectin-3 may decrease NK cell mediated tumor-killing *via* neutrophil ROS release.

Using patient samples from two cohorts of chemo naïve patients diagnosed with HGSC, we detected high levels of Galectin-3 in the tumor metastatic environment of ascites. We also detected high levels of Galectin-3 in HGSC cyst fluid, which is the fluid found surrounding the primary tumor in the ovary. These findings go in line with elevated levels of Galectin-3 reported in many other cancers including colon, head and neck, liver, gastric, endometrial, thyroid, skin and breast carcinomas (25). The Galectin-3 levels in serum or plasma measured in our cohorts were comparable to Galectin-3 serum and plasma levels in healthy subjects measured by us and others (46, 50, 51), indicating that Galectin-3 levels are specifically increased at the primary and metastatic tumor sites. In healthy conditions, Galectin-3 is found in most tissues, including the ovaries, but the expression is low compared to other tissues. Interestingly, Galectin-3 is not found in lymphoid tissues (52). The cellular source of Galectin-3 includes macrophages, neutrophils and epithelial cells (53). In malignant conditions, Galectin-3 can be produced by tumor cells, but also by stimulated lymphocytes. Thus, the increased Galectin-3 levels detected in OC ascites may be a result of increased production in tumor cells as well as increased inflammation in the peritoneum.

In addition to their essential role in host defense against pathogens, neutrophils regulate inflammation and activity of other leukocytes (54–56). In the malignant setting, the role of tumor-associated neutrophils is debated, where neutrophils may both inhibit and promote tumor growth and metastasis (57). High levels of neutrophils have been associated with worse outcome in several cancers including melanoma, renal and lung cancer (58–60), but the knowledge on neutrophils in OC is limited. A recent study suggested that neutrophils might promote OC metastasis, as ovarian tumor cells stimulate the release of NETs, and NETs bound to the tumor cells facilitate metastasis to the omentum (61). Activation of the NADPH oxidase, leading to production of ROS, is an important mechanism for neutrophil elimination of diverse microorganisms and regulation of inflammation (62–64). In addition, because ROS is toxic to tumor cells, the ROS-release by tumor-associated neutrophils may have an anti-tumor effect. However, also NK cells are sensitive to ROS, and addition of histamine dihydrochloride, which inhibits the formation of ROS, spares NK cells from undergoing ROS-induced apoptosis (41, 65). Indeed, immunotherapy with histamine dihydrochloride and low-dose IL-2 leads to improved clinical outcome in acute myeloid leukemia (66, 67).

Our data suggests that a fraction of HGSC patients harbor ascites neutrophils that respond to Galectin-3 with ROS release. It is well established that extracellular Galectin-3 mediates intracellular signaling, which impacts on cell function (24). Depending on the priming status of the neutrophil, different receptors may be ligated by Galectin-3, resulting in various intracellular signaling cascades. *In vitro* stimulation of neutrophils increases Galectin-3 binding proteins on the neutrophil surface, and only extravasated or *in vitro* stimulated neutrophils respond with ROS release upon Galectin-3 exposure (39). Earlier studies have suggested that Galectin-3 binding to CD66a and CD66b on *in vitro* stimulated neutrophils leads to activation of the NADPH oxidase complex (68). However, Galectin-3 binds to β-galactosides present at many different surface receptors, and induces respiratory burst in neutrophils in a carbohydrate- and dose-dependent manner, suggesting that several receptors may be involved in Galectin-3-induced ROS release (69). Generation of extracellular ROS is mediated by the assembly and activation of the NADPH oxidase, which *via* electron transport reduces O_2_ to O_2_^-^. As O_2_^-^ is very unstable, it quickly reacts with protons to form ROS such as H_2_O_2_ and HOCl, by the help of enzymatic reactivity. The signaling cascades that lead to activation of the NADPH oxidase depends on the stimuli; while fMLF induces NADPH oxidase activation via G-protein coupled receptors on the cell surface, PMA stimulates the intracellular protein kinase C (PKC) (70). Both signaling pathways eventually leads to phosphorylation of the NADPH oxidase components, resulting in ROS release. Despite many attempts to understand these complex signaling cascades, not all signaling pathways leading to NADPH oxidase activation are completely identified, including Galectin-3-induced NADPH oxidase activation and ROS release (71).

The fact that only one out of three patients’ neutrophils responded with a low but clear ROS release upon Galectin-3 stimulation suggests that HGSC ascites neutrophils are primed to various extent. Analysis of surface markers demonstrated heterogeneity in degranulation between patients, and all ascites neutrophils could be further degranulated *in vitro*. Thus, the ascites neutrophil priming status varies between different donors as also observed for transmigrated neutrophils in inflamed joints of patients with inflammatory arthritis (72). As neutrophils from different patients varied in degree of degranulation, this may mean that also the level of Galectin-3 binding proteins varied on the neutrophil surface, resulting in differential Galectin-3-induced ROS-responses. The fact that the non-TNF-α treated ascites neutrophils that produced ROS upon Galectin-3 stimulation (patient 30) also were the most degranulated suggests that the extent of degranulation can be of importance for Galectin-3 responsiveness. On the other hand, degranulation is not always equivalent to priming status. In certain circumstances, neutrophils may respond with increased ROS production while they show no or little sign of degranulation (73, 74). For example, treatment with a G protein-coupled receptor (GPCR) internalization inhibitor (Barbadin) and endogenous hyaluronan acid increases GPCR-mediated ROS release without an increase of CD11b or cleavage of CD62L (73, 74). In the OC setting, it is plausible that the transmigration to the peritoneum leads to neutrophil activation. Moreover, ascites contains inflammatory reagents including TNF-α that can activate neutrophils (75). Indeed, we could demonstrate that resting neutrophils from autologous peripheral blood degranulate when exposed to cell-free ascites. In addition, neutrophil incubation in cyst fluid collected at the site of the primary tumor increased surface expression of granule markers and decreased the expression of CD62L. Thus, neutrophils present at the primary tumor site are likely also degranulated and primed.

Another possible explanation for the absence of ROS response to Galectin-3 in some HGSC ascites neutrophils is that they might express surface proteins that affect their response to Galectin-3. For example, Galectin-3C, a truncated version of Galectin-3 that lacks the N-domain but contains the carbohydrate recognition domain (CRD), inhibits Galectin-3-induced ROS release in neutrophils. The levels of Galectin-3C can be increased in situations where high amounts of primed neutrophils are present. Thus, even though additional binding of Galectin-3 to the cell surface of neutrophils is possible (neutrophils reach saturation at around 8 µM, as compared to a maximum of 4.4 nM measured in HGSC ascites in this study and 0.5 nM in healthy donor plasma), it will not induce more ROS production when high amounts of Galectin-3C is bound to the cell surface (40). Additionally, the binding of Galectin-3 in itself may affect the accessibility of surface proteins on the neutrophil as Galectins form lattices by crosslinking glycoproteins on the cell surface of neutrophils. The formation of lattices organizes cell surface-receptors, either by clustering certain receptors, or excluding receptors, thus regulating receptor signaling (76). Nevertheless, we did observe a Galectin-3-induced ROS release in ascites neutrophils from one out of three patients, and all ascites neutrophils responded to Galectin-3 when pre-treated with TNF-α. It is worth noting that the degree of ROS production observed in those experiments is lower when compared to the Galectin-3 response in blood neutrophils observed by others previously (39, 40). Though only observed in two patient samples, we noted a trend that ascites neutrophils responded with higher ROS production upon fMLF stimulation when compared to blood neutrophils. This may imply that ascites neutrophils have higher surface expression of formyl-peptide receptor 1 (FPR1), a receptor for fMLF. FPR1 is exposed on the neutrophil surface when neutrophils extravasate from the blood circulation, or after *in vitro* treatment with agents such as LPS or TNF-α (77–79). Again, these results indicate that ascites neutrophils from some HGSC patients may be in a primed state.

Using a co-culture setup with NK cells and primed neutrophils, we demonstrated that Galectin-3 induces NK cell death in a ROS-dependent manner. Addition of K562 tumor cells to the co-culture evoked NK cell degranulation; however, this was diminished in the presence of ROS produced by neutrophils. These findings suggest that Galectin-3 can contribute to a tumor-promoting environment in which NK cell-mediated tumor eradication is dampened in a neutrophil – Galectin-3-dependent pathway. Due to highly impeded NK cell degranulation when neutrophils were added to the co-culture, we could not detect a further decrease in NK cell degranulation in the presence of Galectin-3. NK cells may also be suppressed by other factors in the complex tumor microenvironment of HGSC; OC ascites contains anti-inflammatory cytokines as well as myeloid-derived suppressor cells (MDSCs) and tumor-associated macrophages (TAMs) (80). A number of studies have shown that MDSCs decrease immune responses leading to increased tumor growth, and using a xenograft tumor model with primary human OC cells, MDSCs were shown to decrease T cell proliferation and anti-tumorigenic properties (81). The presence of cytokines such as IL-10 and TGF-ß, secreted by MDSCs, TAMs and other immune cells, as well as tumor cells, further suppress NK cells (80, 82, 83). For example, TGF-β downregulates the activating receptors NKp30 and NKG2D on NK cells resulting in decreased cytotoxicity (84). Moreover, the presence of soluble B7-H6, a ligand to NKp30, in OC ascites induced downregulation of NKp30, which correlated with impaired anti-tumor function of NK cells (15). Thus, combinatory treatments targeting several immune-evasion pathways in OC should be further investigated.

Galectin-3 may affect NK cell function by causing reduced activation *via* inhibition of activating NK cell receptors. One study reported that Galectin-3 can bind to MICA, which is a ligand to the activating NK cell receptor NKG2D, and that this Galectin-3-MICA complex caused disturbed interaction with NKG2D and thereby reduced NK cell killing of bladder cancer cells (85). It has also been proposed that Galectin-3, by acting as a soluble inhibitory ligand to the activating NK cell receptor NKp30, results in reduced NK cell cytotoxicity towards cervical cancer cells (86). Moreover, Galectin-3 binding to Integrin beta-1 (IGB1; CD29) on NK cells may induce secretion of the anti-inflammatory cytokine IL-10 (87), and Galectin-3 localized intracellularly in NK cells can affect NK cell degranulation, as inhibition of Galectin-3 increased the release of cytotoxic granules (88). Altogether, these studies suggest that Galectin-3 inhibits NK cell mediated tumor killing. However, contrarily to what others have reported, we did not observe decreased NK cell mediated cytotoxicity or decreased degranulation towards K562 target cells in the presence of Galectin-3.

Galectin-3 may be targeted by small molecule inhibitors such as GB0139 or monoclonal antibodies (18). Thus far, targeting Galectin-3 has shown promise for antiviral therapy (18), and Galectin-3-targeting drugs are now being evaluated in both malignant (bladder and colorectal cancer, multiple myeloma, B cell lymphoma, melanoma, chronic lymphocytic leukemia, non-small cell lung cancer) and non-malignant (fibrosis, non-alcoholic steatohepatitis, psoriasis) conditions (25). We have recently reported that NK cells present at the ascitic metastatic site in HGSC demonstrate anti-tumor capacity (7); however, further interventions may be required to overcome the immunosuppressive environment in ascites. Indeed, Galectin-3 is suggested as an immunotherapeutic target improving the outcome of inhibitory checkpoint inhibition in cancer immunotherapy, were Galectin-3 inhibition for example was demonstrated to augment the PD-L1 response in a mouse model of lung adenocarcinoma (89, 90). The results from this study demonstrate a ROS mediated decrease of the NK cell anti-tumor response. Thus, another therapeutic approach may be to inhibit ROS formation through treatment with histamine dihydrochloride as proven efficient in acute myeloid leukemia (66). Even though our studies have focused on the metastatic site of HGSC ascites, we also show that Galectin-3 levels are high in HGSC cyst fluid at the primary tumor site, and that neutrophils are degranulated when incubated in HGSC cyst fluid. Ultimately, these results suggest that Galectin-3 may affect NK cell mediated anti-tumor response also at the primary tumor site.

Murine models that mimic human conditions, such as xenograft mouse models, can generate valuable information about human biology that is challenging to obtain otherwise. Using a murine model to study if Galectin-3 induces neutrophil-dependent ROS mediated NK cell death and reduced cytotoxicity towards OC tumor cells *in vivo* could therefore be of interest for future perspectives. However, there are substantial differences between human and murine neutrophils, including receptor signaling pathways, granule proteins and regulation of NADPH oxidase activity (91), which have to be taken into consideration.

A limitation with this study is the low number of samples in certain experiments, including measurement of ROS activity. Nevertheless, despite a low number of samples we believe that this study provides new insights into the interplay between NK cells, neutrophils and Galectin-3.

In conclusion, we report that the high levels of Galectin-3 detected in the tumor microenvironment of HGSC may decrease NK cell eradication of tumor cells in a ROS-dependent manner. This study sheds light on the intricate immune cell interactions within the tumor microenvironment in OC and suggests further investigation to evaluate Galectin-3 as a potential immunotherapeutic target in OC.

## Data Availability

The original contributions presented in the study are included in the article/**Supplementary Material**. Further inquiries can be directed to the corresponding author.
