# Supplementary material for: Elevated Galectin-3 levels in the tumor microenvironment of ovarian cancer – implication of ROS mediated suppression of NK cell antitumor response via tumor-associated neutrophils
Source: Front Immunol. 2024 Dec 20;15:1506236. doi: 10.3389/fimmu.2024.1506236 (PMC11695286; doi:10.3389/fimmu.2024.1506236)
Supplement: Supplementary file 1 [file DataSheet1.pdf]

Supplementary Material

1 Supplementary Data

2 Supplementary Figures and Tables

2.1 Supplementary Figures

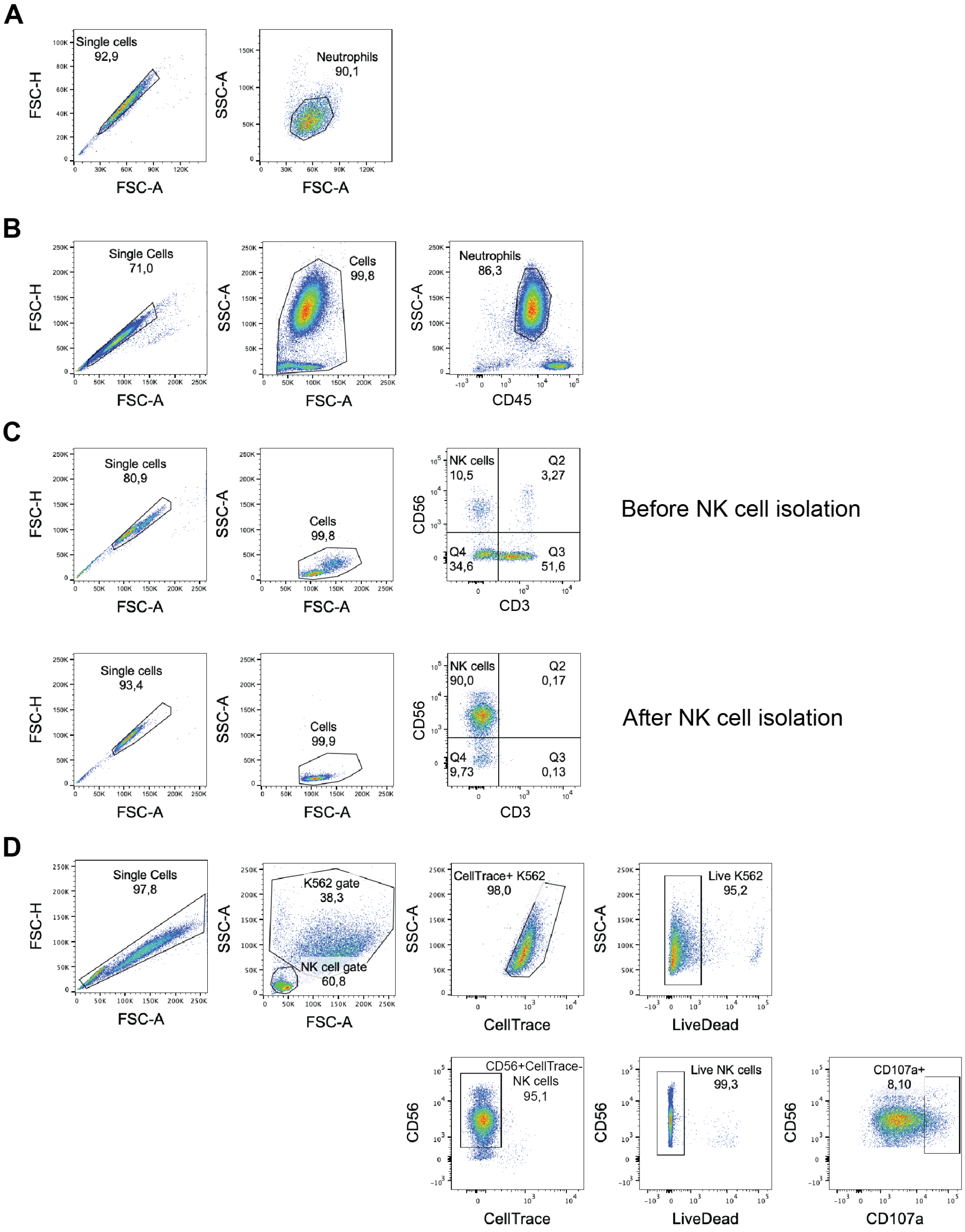

Supplementary Figure S1. Cell identification and gating strategies used in flow cytometry experiments. (A) Neutrophils isolated from ascites and blood were identified by side scatter (SSC) and forward scatter (FSC). (B) Neutrophils were identified by SSC and CD45 expression in filtrated ascites and blood. (C) Prior to functional assays, NK cells were identified as $CD56^{+}CD3^{-}$ cells in peripheral blood mononuclear cells before and after NK cell isolation. (D) In functional assays, K562 cells were identified as $CellTrace^{+}$ cells, and NK cells were identified as $CD56^{+}$ $CellTrace^{-}$ cells. Cell viability was measured using LIVE/DEAD Fixable Near-IR Dead Cell Stain Kit (Invitrogen), and NK cell degranulation was evaluated using a CD107a antibody.

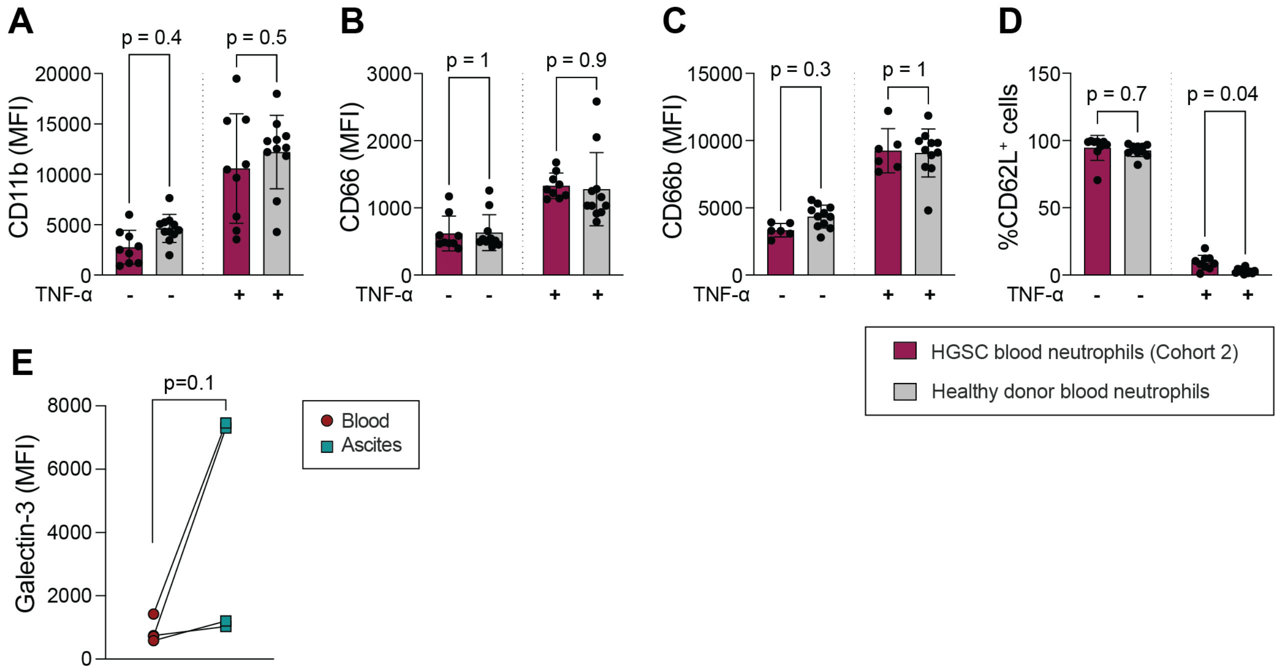

Supplementary Figure S2. Surface expression of CD11b (A), CD66 (B), CD66b (C), and percentage of CD62L$^+$ neutrophils (D) on unstimulated and TNF-α treated blood neutrophils isolated from patients with HGSC or healthy donors. Data is presented as mean ± SD and ordinary one-way ANOVA followed by Šídák's multiple comparisons test was used for statistical analysis (n=6-9 HGSC, n=11 healthy donors). (E) Level of surface-bound Galectin-3 on paired peripheral blood and ascites neutrophils. Paired Student's t-test was used for statistical analysis (n=4).

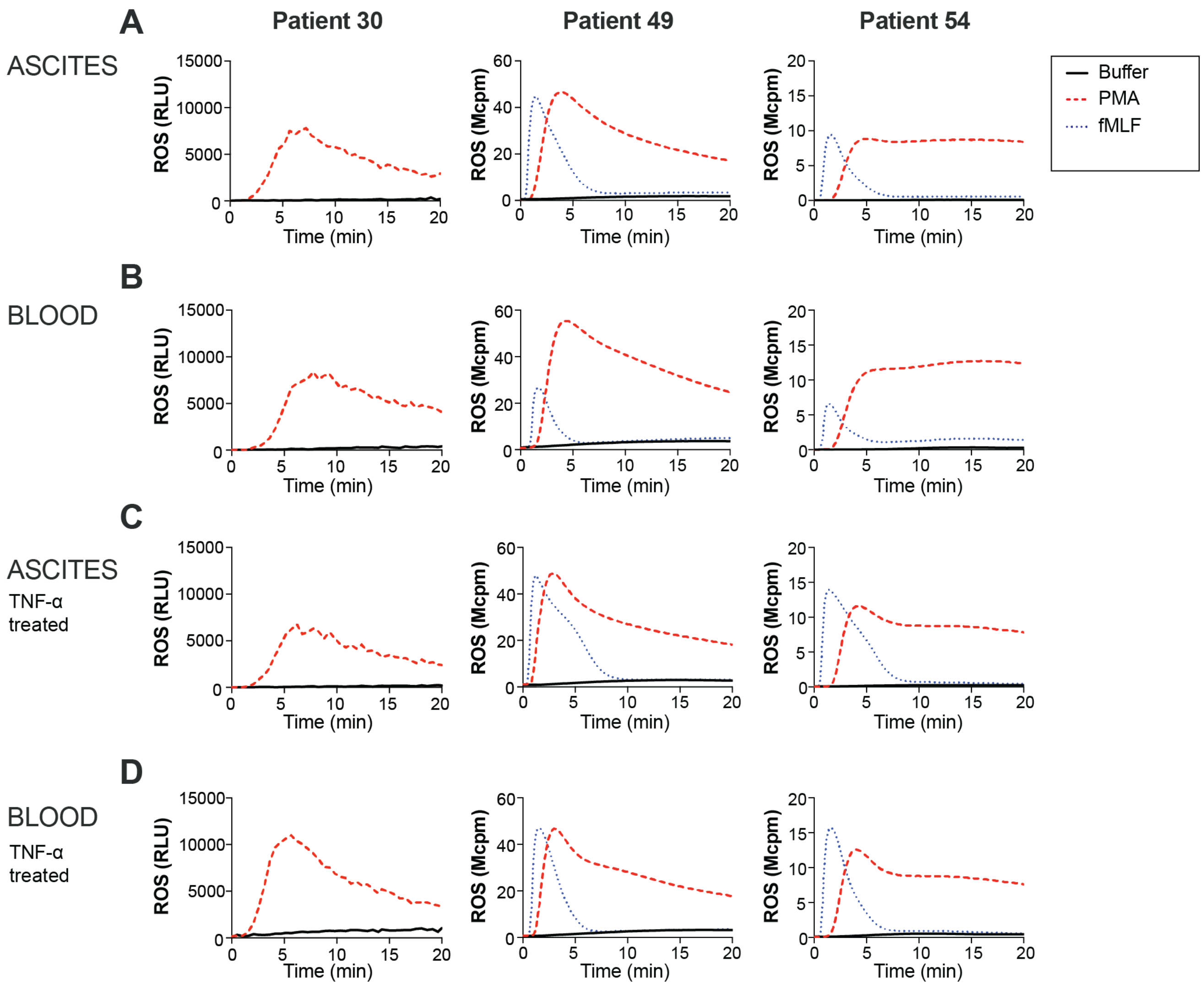

Supplementary Figure S3. ROS release in ascites and peripheral blood neutrophils from 3 HGSC patients. (A-D) ROS release upon exposure to fMLF or PMA in unstimulated ascites (A) or blood (B) neutrophils, and TNF-α treated ascites (C) or blood (D) neutrophils. For patient 30, neutrophils were not stimulated with fMLF. ROS release in neutrophils from patient 30 was measured using CLARIOstar plate reader, while ROS release in neutrophils from patient 49 and 54 was measured using Biolumat LB 9505.

2.2 Supplementary Tables

Supplementary Table S1. Age and Galectin-3 serum levels in healthy donors.

Patient ID	Age (in years)	Serum Galectin-3 concentration (ng/mL)
**2**	52	6.8
**4**	57	9.3
**7**	59	7.0
**8**	46	10.6
**10**	68	12.3
**13**	54	10.8
**15**	58	11.9
**16**	65	11.1
**17**	70	8.1
**18**	61	8.0
**19**	67	14.2
**20**	59	8.3
**21**	52	8.9
**26**	51	10.7
**27**	51	7.7
**28**	46	8.6
**29**	63	21.9
**45**	45	8.9
